# Medical student sensitivity training on the differences in sex development

**DOI:** 10.5116/ijme.631e.e8fd

**Published:** 2022-09-28

**Authors:** Paul Endres, Deborah Ziring, Dimitrios Papanagnou

**Affiliations:** 1 Sidney Kimmel Medical College, Thomas Jefferson University, Philadelphia, USA; 2 Department of Emergency Medicine, Sidney Kimmel Medical College, Thomas Jefferson University, Philadelphia, USA

**Keywords:** Sex development, LGBTQ+ health, medical training, Intersex education, medical education

## To the Editor

LGBTQ+ health concerns have become increasingly prevalent in medical education. However, intersex individuals and their respective healthcare needs are frequently lumped into this category and not appropriately represented as standalone content in standard curricula. Intersex education is typically taught in conjunction with disorders of sexual development – an outdated and stigmatized term. The authors, who represent medical student and faculty educator stakeholders, aimed to develop a discrete intersex educational pilot training session to better inform how conversations supporting intersex individuals can be facilitated in undergraduate medical education.

Over the last decade, medical schools have rightfully expanded curricular content to educate students on LGBTQ+ health concerns, prompting students to ask for patient pronouns and take a more holistic sex history with sensitivity.^1^^, ^^2^ The care of intersex individuals, however, is frequently consolidated into standard LGBTQ+ training and, as a result, is not appropriately represented in medical education curricula as a standalone curricular topic.^3^ Specifically, intersex education is frequently taught in conjunction with disorders of sex development (DSD) - an outdated and stigmatized term. This approach minimizes the intersex experience and does not address the current controversy surrounding DSDs, which are now more commonly described as differences in sex development, to de-stigmatize and de-pathologize intersex bodies.^4^ The authors suggest a more intentional approach to intersex education in order to train clinicians to provide patient-centered, trauma-informed care for this subset of the patient population and address their respective healthcare needs.

While the actual incidence rate of differences in sex development remains unclear, it is estimated to be 1 in 4,500-5,500.^5^ Currently, the Intersex Society of North America reports there is a total of 1 in 100 people whose bodies differ from standard male and female.^6^ In the medical education literature, being an intersex individual is presented as part of the LGBTQ community. In terms of education and training materials available to the community, when searching intersex in the medical education literature available online, there is a paucity of resources available to guide the medical education community – and most of which pertain to general LGBTQ+ education. The education community requires standalone educational materials and training resources focusing on intersex education. To this effect, there is a need to reframe the care of the intersex community that respects this unique community's individuality while using appropriate language in order to create a safe space that allows intersex patients to feel supported and empowered as they interact with healthcare providers.

At our institution, our student-run LGBTQ affinity group (referred to as JeffLGBTQ of the Sidney Kimmel Medical College at Thomas Jefferson University in Philadelphia, Pennsylvania, United States of America) was prompted to respond to this educational gap and advocate for additional training opportunities. JeffLGBTQ is well known at our institution for providing informative and educational sessions surrounding a number of different genders and sexual minority topics. Pre-clinical medical students, in particular, felt that the intersex community was marginalized in lectures surrounding DSD and intersexuality. Students felt that the content was not fully encompassing the history of intersex individuals and that the language was outdated. By engaging with intersex individuals, intersex advocates, and faculty stakeholders in the medical school, JeffLGBTQ organized an educational session entitled Intersex 101, to help educate students on the history, language, and concerns of people living with intersex conditions. Knowing the history of intersex medical care, in turn, has the potential to prepare them to be more patient-centered and take a trauma-informed approach to serve this population. A trauma-informed approach focuses on safety, empowerment, collaboration, and support. This model shifts the lens of care from "what is wrong with you?" to one that is more aligned with "what has happened to you?" This shift is critical as many of these individuals may have experienced traumatic medical care.

This session was designed to complement content in our undergraduate medical education curriculum as a standalone extracurricular offering. It was open to all members of our institution's community, including students, residents, and faculty. No previous training was required to attend. The invited facilitator was a urologist and intersex advocate with expansive knowledge serving the intersex community from her work with interACT. InterACT is the world's largest intersex advocacy organization, which advocates for the human rights of children born with intersex traits. To encourage attendance, the session was delivered remotely over Zoom. We were also fortunate to have the insight of another member of interACT who identifies as an intersex individual to provide their experiences of their medical care. The session emphasized four topics to our learners – empathy, engagement, language, and education.

First and foremost, we aimed to discuss the experiences of individuals living with differences in sexual development. We stressed the power of language as the words we use as health providers are powerful. By using the word difference, in lieu of disorder, we are creating a space that does not stigmatize an individual while still being accurate to the condition they are living with.  The session was attended by medical students, residents, and faculty. Clinical faculty asked insightful questions surrounding the intersex experience and how to best inform their practice of medicine, with specific regards to obstetrics, gynecology, and urology. Medical students engaged with the invited intersex panelist, who was able to speak personally about their lived intersex experience. One faculty member specifically shared that she appreciated how "patient-driven care can reorient us to our patient's goals for their anatomy and how we can help them meet those needs." Despite our advertising and the high level of engagement in our attendees, attendance for this voluntary session as low – especially when considering our medical student body of approximately 1100 students. Sessions such as these should not be voluntary; instead, they should be part of a formal curriculum.

It is important to learn about this community independently and on its own, as members of this community have their own unique treatment challenges, including a common history of medical trauma. We are thankful we had a facilitator and community member, both of whom were able to speak on these topics openly and authentically. As this was an optional session and not required, we are limited in our ability to evaluate its longitudinal impact fully. Our session is at the forefront of a wave of inclusivity in medical education. As our society grows in its understanding of sex and sex development, we call upon medical education leaders to think critically about more effective ways to teach sex development to clinicians in training. We hope our efforts will inform and expand curricular opportunities that better prepare trainees to interact with individuals with differences in sex development with empathy, respect, and engagement.

### Conflicts of Interest

The authors declare that they have no conflict of interest.
